# Free Medical University

**Published:** 1857-08

**Authors:** 


☞ We are requested to state to the profession, through the Journal, that in consequence of the abolition of fees by the Trustees of the Medical Department of Iowa University, making the course of instruction *free to all*, no scholarships are needed and none will be issued. It is hoped that the friends of the school will note the fact, and advise students accordingly. Scholarships, if received, will be those issued for the purpose of entrapping the unwary by those having no connection whatever with the school.

Circulars will be distributed within a few days, fully setting forth the position of affairs. We may say to those interested in the welfare of the institution that letters of inquiry are constantly coming to hand from those wishing to attend the session of 1857 and '58, and there is every indication of a larger class than ever before. W. R. M.

